# Glycine Attenuates Lipopolysaccharide-Induced Acute Lung Injury by Regulating NLRP3 Inflammasome and NRF2 Signaling

**DOI:** 10.3390/nu12030611

**Published:** 2020-02-26

**Authors:** Yunchang Zhang, Xiaoshi Ma, Da Jiang, Jingqing Chen, Hai Jia, Zhenlong Wu, In Ho Kim, Ying Yang

**Affiliations:** 1 State Key Laboratory of Animal Nutrition, Department of Animal Nutrition and Feed Science, China Agricultural University, Beijing 100193, China; zycforward@hotmail.com (Y.Z.); maxiaoshi0310@163.com (X.M.); jayd1994@163.com (D.J.); CJQ9512@163.com (J.C.); jiahai1992@163.com (H.J.); bio2046@hotmail.com (Z.W.); 2 Beijing Advanced Innovation Center for Food Nutrition and Human Health, China Agricultural University, Beijing 100193, China; 3 Department of Animal Resource & Science, Dankook University, Cheonan 330-714, Korea; inhokim@dankook.ac.kr

**Keywords:** acute lung injury, glycine, HSP70, lipopolysaccharide, NF-κB, NLRP3, NRF2

## Abstract

Glycine supplementation has been reported to alleviate lipopolysaccharide (LPS)-induced lung injury in mice. However, the underlying mechanisms responsible for this beneficial effect remain unknown. In the present study, male C57BL/6 mice were treated with aerosolized glycine (1000 mg in 5 mL of 0.9% saline) or vehicle (0.9% saline) once daily for 7 continuous days, and then were exposed to aerosolized LPS (5 mg in 5 mL of 0.9% saline) for 30 min to induce lung injury. Sera and lung tissues were collected 24 h post LPS challenge. Results showed that glycine pretreatment attenuated LPS-induced decreases of mucin at both protein and mRNA levels, reduced LPS-triggered upregulation of pro-inflammatory cytokines, such as tumor necrosis factor-α (TNF-α), interferons, granulocyte-macrophage colony-stimulating factor (GM-CSF), and interleukins. Further study showed that glycine-reduced LPS challenge resulted in the upregulation of nuclear factor κB (NF-κB), nucleotide binding domain (NOD)-like receptor protein 3 (NLRP3) inflammasome. In addition, LPS exposure led to the downregulation of NRF2 and downstream targets, which were significantly improved by glycine administration in the lung tissues. Our findings indicated that glycine pretreatment prevented LPS-induced lung injury by regulating both NLRP3 inflammasome and NRF2 signaling.

## 1. Introduction

Acute lung injury is a common and severe pulmonary complication of critical illness caused by multiple factors, such as pneumonia, sepsis, shock, and respiratory bacteria or viruses infection [1]. Under physiological conditions, an intracellular homeostasis and a normal function of the lungs are maintained by the host defense systems, including mucus layer and immune response. Mucus layer, which is mainly consisted of secreted mucins, including MUC5AC and MUC5B, is the first defense line that prevents the contact of host cells with lumen contents, such as chemicals, toxins, and pathogens [2]. The dysfunction of mucus is associated with bacteria invasion, which in turn activates immune response, infiltration of macrophage, and the secretion of pro-inflammatory cytokines, such as tumor necrosis factor-α (TNF-α), interleukin-1β (IL-1β), and interferon-γ (IFN-γ), leading to decline of lung function and the manifestation of tissue damage [3,4].

An increased level of lipopolysaccharides (LPS), a cell wall component of Gram-negative bacteria, has been observed in the plasma and lung tissues of clinical patients and experimental animals. Consistently, a decreased LPS level is associated with a positive treatment outcome and a better prognosis, indicating an important role of LPS in the development of lung diseases. A series of pathogen recognition receptors (PRRs), such as Toll-like receptors (TLR), RIG-I-like receptors (RLR), protease-activated receptors (PAR), Nod-like receptors (NLR), C-type lectin receptors, have been reported to sense the pathogens in contact with the airway epithelium and activate host response [5]. Several lines of evidence indicates that LPS can be recognized by TLR4, and induces the accumulation of myeloid differentiation factor 88 (MYD88), which ultimately leads to the activation of nuclear factor κB (NF-κB)—a critical transcriptional factor implicated in producing of pro-inflammatory cytokines [6]. The nucleotide-binding domain-like receptor protein 3 (NLRP3) inflammasome is a major intracellular multiprotein complex of the innate immune system, which is composed of a NOD-like receptor, NLRP3 protein, apoptosis-associated speck-like protein (ASC) and procaspase1 [7]. NLRP3 inflammasome has been reported to play an essential role in the inflammation responses during acute lung injury [8,9]. Consistently, NLRP3-deficient mice are resistant to bacteria-induced lethality [9,10,11], suggesting an important regulator of lung diseases [12].

In addition to inflammatory responses, the host cells have multiple signaling pathways, whose activation is associated with cell survival in response to various stimuli. The nuclear factor erythroid-2 related factor 2 (NRF2) is a transcriptional factor implicated in inflammation- and oxidative stress-related diseases [13]. Activation of NRF2 upregulates down-stream targets, such as heme oxygenase 1 (HO1), NAD(P)H quinone dehydrogenase 1 (NQO1), and glutathione s-transferase α 4 (GSTA4) [14], to reduce antioxidative damage and promotes detoxification in multiple pulmonary diseases [15,16]. Deletion of NRF2 is associated with high susceptibility to respiratory bacterial infection [17].

Despite enormous advances in treating lung injuries, therapeutic options with high efficacy and less side effect are imperative, considering its high morbidity and mortality all over the world [18]. Amino acids, such as glutamine, arginine, and glycine, have attracted more and more attention due to various bioactivities, such as anti-inflammatory and anti-oxidative effects, in the lung and other tissues [19,20,21,22]. Our recent study has showed that glycine pretreatment effectively alleviated LPS-induced lung injury by inhibiting the inflammation and apoptosis of alveolar cells [23]. Nevertheless, the underlying mechanism still remains unclear. The objective of the present study was to test the hypothesis that glycine supplementation attenuated LPS-induced acute lung injury by regulating NLRP3 inflammasome and NRF2 signaling.

## 2. Materials and Methods

### 2.1. Reagents

LPS (*Escherichia coli* O55:B5) and glycine were products of Sigma (St. Louis, MO, USA). Alcian blue (pH 2.5) staining kit was obtained from Vector Laboratory (Burlingame, USA). Commercial mouse inflammation panel kit was purchased from BioLegend (San Diego, USA). Primers were synthesized by Sangon Biotech Co. (Shanghai, China). Total RNA extraction kit was purchased from Aidlab Biotechnologies (Beijing, China). FastQuant RT Kit (with gDNase) and SuperReal PreMix Plus (SYBR Green) were purchased from TIANGEN Biotech (Beijing, China). Antibodies against GAPDH, Actin, phosphor-P65 (p-P65), NLRP3, Procaspase1, Cleaved caspase1, NRF2, and Beclin1 were purchased from Santa Cruz Biotechnology (Santa Cruz, CA, USA). Antibodies against P65, P62, autophagy related gene 5 (ATG5), and microtubule-associated protein light chain 3 (LC3) were products of Cell Signaling Technology (Danvers, MA, USA). Antibodies against TLR4, MYD88, ASC, HO1, NQO1, GSTA4, HSP40, HSP70, and HRP-conjugated rabbit anti-mouse, and mouse anti-rabbit secondary antibodies were gained from Sangon Biotech Co. (Shanghai, China).

### 2.2. Experimental Design

All experiments were approved by the Animal Care and Use Committee of China Agricultural University, and conformed to the Guide for the Care and Use of Laboratory Animals.

A total of 21 male C57BL/6 mice weighed 18 ± 2 g (Huafukang Biotechnology Ltd, Beijing, China) were housed in a room with a temperature of 23 ± 1 °C and a 12-h light/12-h dark cycle. Mice had free access to feed (Huafukang Biotechnology Ltd, Beijing, China, No. 1022) and drinking water. After a 1-week adaptation period, mice were randomly assigned to one of three treatment groups: control group (CON), LPS treatment group (LPS group), and glycine pretreatment + LPS treatment group (Gly + LPS group). Mice in the CON group were exposed to aerosolized 0.9% saline (5 mL), while mice in Gly + LPS treatment group or LPS treatment group were exposed to aerosolized glycine (1000 mg in 5 mL of 0.9% saline) or equal volume of aerosolized 0.9% saline once daily for 7 continuous days, based on our pilot study and previous report [24]. Then, mice in LPS or Gly + LPS treatment group were exposed to aerosolized LPS (5 mg in 5 mL of 0.9% saline) for 30 min on the 8^th^ day of the experiment. Mice were sacrificed 24 h after LPS exposure. Orbital blood was collected and serum was harvested for cytokines analysis. Right lung lobes were dissected, washed in pre-cooled phosphate buffer saline (PBS), and then were stored at −80 °C for later analysis.

### 2.3. Alcian Blue Staining 

Lung tissues fixed in 4% formaldehyde were dehydrated, embedded in paraffin, sectioned, and stained with alcian blue solution (blue) and nuclear fast red solution (red) according to manufacturer’s instructions (Burlingame, USA). Sections from each mouse were visualized by a blinded observer and pictured using a light microscope equipped with a computer-assisted morphometric system.

### 2.4. Serum Inflammatory Cytokine Analysis

Serum inflammatory cytokines were detected using a commercial mouse inflammation panel kit (BioLegend, San Diego, USA) and the CytExpert Flow Cytometer (Beckman, USA) according to the instructions of manufacturer. Data were analyzed by the LEGENDplex™ software (BioLegend, San Diego, USA).

### 2.5. Quantitative Real-Time Polymerase Chain Reaction (qRT-PCR)

Total RNA was extracted from the lung tissues using a TRIzol kit according to the instructions. The cDNA was obtained by reverse transcription of total RNA, which was carried out by a FastQuant RT Kit (with gDNase). RNA concentration and value of OD260/280 were measured using the Nanodrop P330 (Implen, Germany). The integrity of total RNA with an OD260/280 value of 1.8–2.0 was assessed by 1% agarose gel electrophoresis before performing qPCR experiments. qRT-PCR was performed by the SYBR green mix and specific primers for target genes with the ABI-Prism 7500 Sequence Detection System (Applied Biosystems) according to the instructions of manufacturer. The primer sequences used in the present study are listed in Table 1. *Gapdh* was used as an internal control. The 2^−ΔΔCT^ method was used to determine the fold changes of mRNA levels with the Microsoft Excel software.

### 2.6. Western Blotting

Lung tissues were homogenized in liquid nitrogen, dissolved and vortexed in cold radio-immunoprecipitation assay (RIPA) buffer (10 mm Tris-HCl, pH 7.4; 150 mm NaCl; 10 mm EDTA; 1% NP-40; 0.1% SDS) for protein extraction. Protein abundance was determined by using the Western blotting technique, as described previously [25]. The protein bands were developed by a chemiluminescence kit (Amersham Biosciences) using the Image Quant LAS 4000 mini system (GE Healthcare Bio-sciences). Protein band density was quantified by the ImageJ software (GE Healthcare Life Sciences). 

### 2.7. Statistical Analysis

All data are presented as means ± SEM. Results were analyzed by 1-way ANOVA, using the SAS software, version 9.1 (SAS Institute Inc., Cary, North Carolina, USA.). Differences between means were determined by using the Student–Newman–Keuls multiple-comparison test. *p* < 0.05 was taken to indicate statistical significance.

## 3. Results

### 3.1. Glycine Supplementation Restored Mucin Layer in LPS-Treated Mice

**Figure 1:**
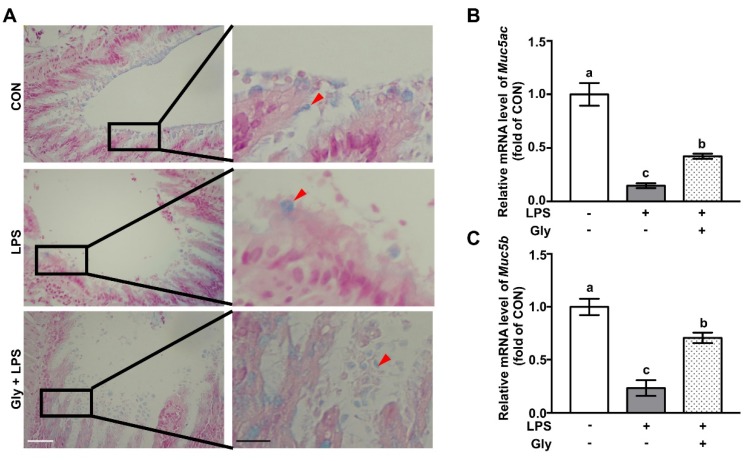
Glycine supplementation restored mucin in LPS-treated mice. (**A**) Alcian blue staining; (**B**,**C**) relative mRNA levels of *Muc5ac* and *Muc5b.* Red arrows indicate goblet cells. White and black scale bars mean 50 and 20 μm respectively. The mRNA level in the CON group was set as 1.00 to calibrate the relative levels. *Gapdh* was used as a reference gene. Values are means ± SEM, *n* = 7. Means for an indicated parameter without a common letter differ, *p* < 0.05.

Compared with the controls, LPS exposure led to reduced airway mucin at protein level as shown by alcian blue staining, which was remarkably restored by glycine (Figure 1A). qRT-PCR analysis showed that mice in the LPS treatment group had significant downregulation of *Muc5ac* and *Muc5b* at the mRNA level in the lung tissues, as compared with the controls (Figure 1B,C). However, glycine supplementation significantly increased (*p* < 0.05) the mRNA levels of *Muc5ac* and *Muc5b,* as compared with those of mice in the LPS treatment group (Figure 1B,C).

### 3.2. Glycine Pretreatment Suppressed Secretion of Pro-Inflammatory Cytokines

**Figure 2:**
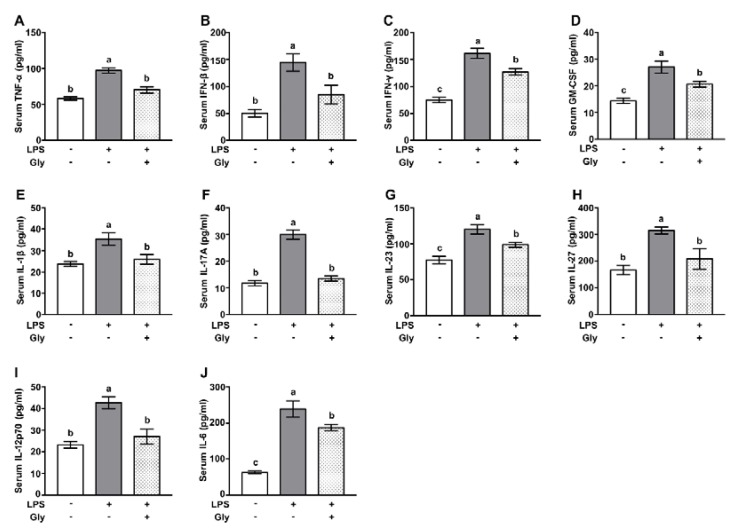
Glycine suppressed secretion of pro-inflammatory cytokines. (**A**–**J**) Serum protein levels of TNF-α, IFN-β, IFN-γ, GM-CSF, IL-1β, IL-17A, IL-23, IL-27, IL-12p70, and IL-6. Values are means ± SEM, *n* = 7. Means for an indicated parameter without a common letter differ, *p* < 0.05.

Mice exposed to aerosolized LPS had increased protein levels of TNF-α, IFN-β, IFN-γ, and granulocyte-macrophage colony-stimulating factor (GM-CSF) in serum, relative to the controls. Glycine administration significantly suppressed the increases of these inflammatory cytokines (Figure 2A–D) (*p* < 0.05). In addition, as compared with the controls, aerosolized LPS exposure led to increased (*p* < 0.05) protein level of IL-1β, IL-17A, IL-23, IL-27, IL-12p70, and IL-6 in serum (Figure 2E–J)**,** which were largely reversed by glycine supplementation (*p* < 0.05).

### 3.3. Glycine Inhibited Activation of NF-κB in LPS-Stimulated Lung

**Figure 3:**
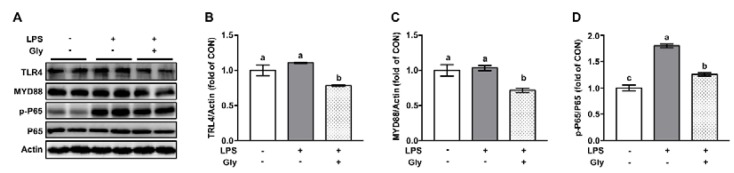
Glycine inhibited activation of NF-κB in lung tissue of LPS-challenged mice (**A**) Representative protein bands of TLR4, MYD88, p-P65, P65, and Actin; (**B**–**D**) statistical analysis of protein abundance. The quantification of band in the CON group was set as 1.00 to calibrate the relative levels. Actin was used as a loading control. Values are means ± SEM; *n* = 7. Means without a common letter differ, *p* < 0.05.

Western blotting results showed that LPS treatment led to increased (*p* < 0.05) phosphorylation of P65, without affecting that of TLR4 and MYD88 at protein level (Figure 3**)**. LPS-induced activation of P65 was significantly suppressed by glycine pretreatment. Of note, protein levels of TLR4 and MYD88 in the lung tissues of mice treated with aerosolized LPS plus glycine were lower (*p* < 0.05) than that of mice in the control or LPS treatment group (Figure 3A–C).

### 3.4. Glycine Pretreatment Blocked Activation of NLRP3 Inflammasome in Lung Tissues of LPS-Challenged Mice 

**Figure 4:**
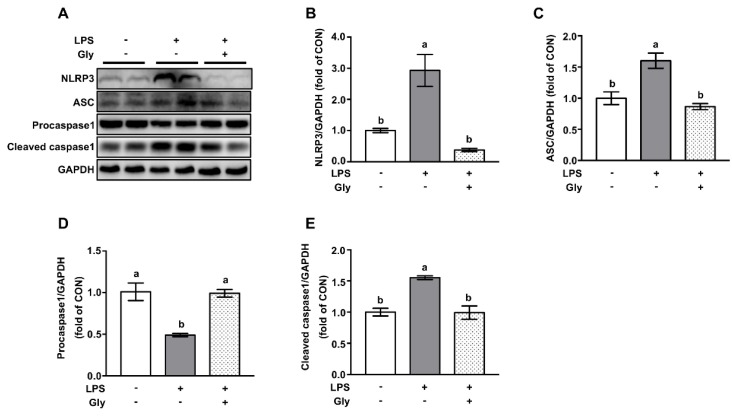
Glycine inhibited activation of NLRP3 inflammasome in lung tissues of LPS-challenged mice. (**A**) Representative protein bands of NLRP3, ASC, Procaspase1, Cleaved caspase1, and GAPDH; (**B**–**E**) statistical analysis of protein abundance. The quantification of band in the CON group was set as 1.00 to calibrate the relative levels. GAPDH was used as a loading control. Values are means ± SEM; *n* = 7. Means without a common letter differ, *p* < 0.05.

Western blotting results showed that LPS treatment increased (*p* < 0.05) the protein levels of NLRP3, ASC, and cleaved caspase1—a downstream target of NLRP3—and decreased the protein level of procaspase1 in comparison to the controls, indicating the activation of the NLRP3 inflammasome (Figure 4). This effect of LPS was remarkably prevented (*p* < 0.05) by glycine pretreatment.

### 3.5. Glycine Administration Enhanced NRF2 Signaling in Lung Tissues of LPS-Challenged Mice 

**Figure 5:**
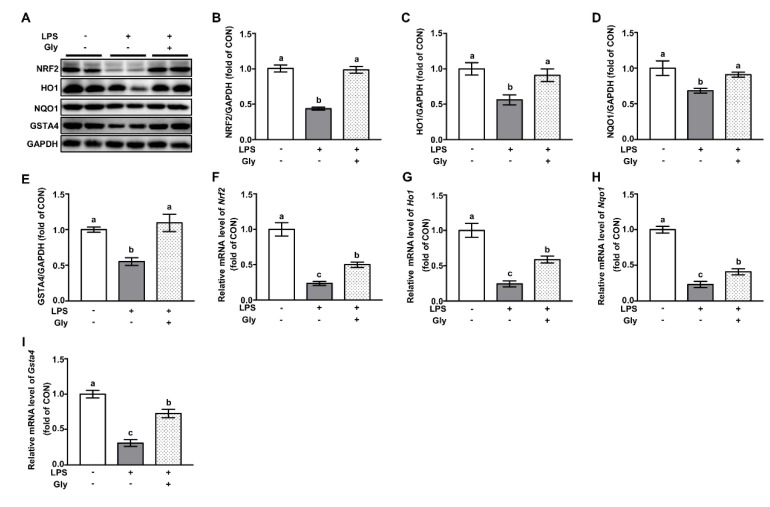
Glycine abrogated LPS-induced NRF2 downregulation in lung tissues of LPS-challenged mice. (**A**) Representative protein bands of NRF2, NQO1, HO1, GSTA4, and GAPDH; (**B**–**E**) statistical analysis of protein abundance; (**F**–**I**) relative mRNA levels of *Nrf2*, *Ho1*, *Nqo1*, and *Gsta4.* The protein abundance or mRNA level in the CON group was set as 1.00 to calibrate the relative levels. GAPDH was used as an internal control. Values are means ± SEM; *n* = 7. Means without a common letter differ, *p* < 0.05.

Compared with the controls, LPS treatment resulted in decreased (*p* < 0.05) protein abundance of NRF2, and down-stream targets, such as HO1, NQO1, and GSTA4, which were abolished by glycine (Figure 5A–E). qRT-PCR results showed that *Nrf2* gene expression was down-regulated in response to LPS exposure, along with downstream targets, such as *Ho1*, *Nqo1*, and *Gsta4*. However, these effects of LPS were attenuated (*p* < 0.05) by glycine supplementation (Figure 5F–I).

### 3.6. Glycine Increased Protein Abundance of HSP70 and HSP40 in Lung Tissues of LPS-Challenged Mice 

**Figure 6:**
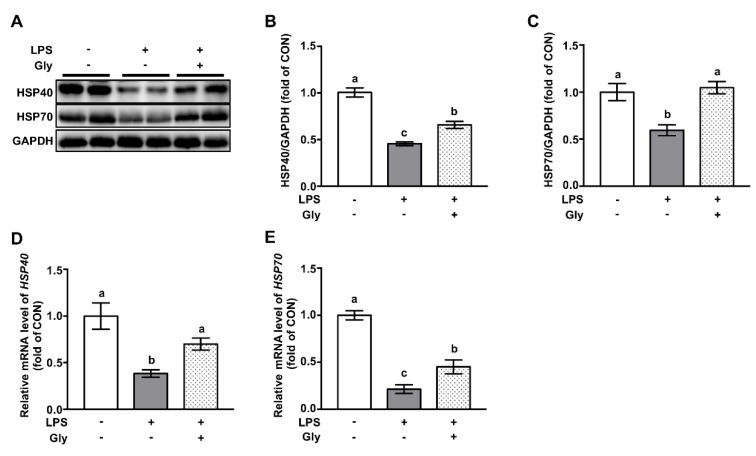
Glycine attenuated LPS-induced downregulation of HSP70 and HSP40 in lung tissues of LPS-challenged mice. (**A**) Representative protein bands of HSP40, HSP70, and GAPDH; (**B**,**C**) statistical analysis of protein abundance; (**D**,**E**) relative mRNA levels of *Hsp40* and *Hsp70.* The protein abundance or mRNA level in the CON group was set as 1.00 to calibrate the relative levels. GAPDH was used as an internal control. Values are means ± SEM; *n* = 7. Means for an indicated parameter without a common letter differ significantly, *p* < 0.05.

Western blotting results indicated that LPS exposure led to the decreased protein abundance of HSP40 and HSP70, which were reversed (*p* < 0.05) by glycine administration (Figure 6A–C). qRT-PCR results showed that mRNA levels of *Hsp40* and *Hsp70* were reduced by LPS treatment, as compared with that of controls, which were significantly prevented by glycine supplementation (*p* < 0.05) (Figure 6D,E).

### 3.7. Autophagy was not Involved in the Beneficial Effect of Glycine on LPS-Induced Acute Lung Injury

**Figure 7:**
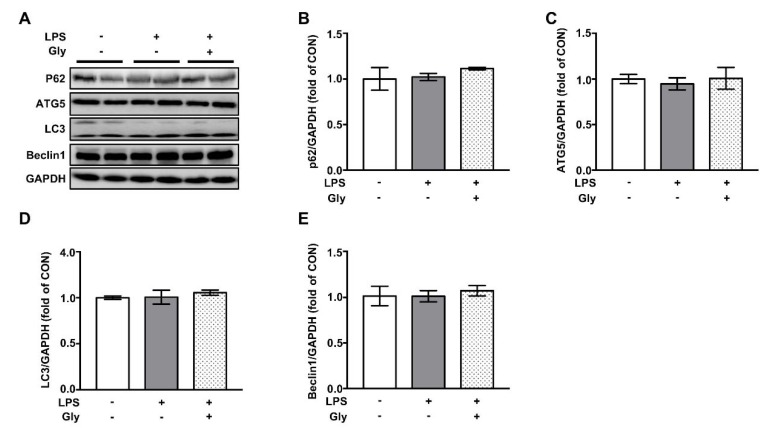
The protein abundance of autophagy markers in in lung tissues of LPS-challenged mice. (**A**) Representative protein bands of P62, ATG5, LC3, Beclin1, and GAPDH; (**B**–**E**) statistical analysis of protein abundance. The quantification of band in the CON group was set as 1.00 to calibrate the relative levels. GAPDH was used as a loading control. Values are means ± SEM; *n* = 7. Means without a common letter differ, *p* < 0.05.

As shown by the Western blotting results, the inhalation of aerosolized LPS had no effect on abundance of protein involved in autophagy, such as P62, ATG5, LC3, and Beclin1 (Figure 7). Furthermore, pre-treatment with glycine did not affect autophagic proteins expression.

## 4. Discussion

In the present study, we found that glycine prevented aerosolized LPS exposure-induced reduction of mucin and upregulation of pro-inflammatory cytokines. This beneficial effect is associated with the inhibition of the NF-κB and NLRP3 inflammasome signaling pathway, as well as the restoration of NRF2 signaling.

The exposure of airway epithelium to respiratory pathogens, allergens, and toxins is associated with epithelial dysfunction and the development of lung diseases. Mucin produced by goblet cells, airway epithelial cells, and mucous cells in submucosal glands is one of the main components of a defense line that covers the air way cells, therefore preventing the contact of lumen contents with epitheliums [26]. The deregulation of the mucus barrier has been reported to be associated with lung injury and the pathogenesis of multiple lung diseases [27]. In the present study, mice pretreated with glycine were subjected to aerosolized LPS, which has been previously reported to induce lung injury [23]. We found that glycine administration prevented the LPS-induced downregulation of mucin at both protein and mRNA levels, which was consistent with previous studies [28,29]. This reduction of mucin following LPS treatment facilitate contact of risk factors with epithelial cells, thus contributing to the dysfunction of the respiratory barrier and inflammatory responses [30,31,32]. As expected, mice treated with LPS had elevated protein levels of proinflammatory cytokines such as TNF-α, IFN-β, IFN-γ, GM-CSF, and interleukins, such as IL-1β, IL-17A, IL-23, IL-27, IL-12p70, and IL-6 in serum, indicating the occurrence of lung injury in response to aerosolized LPS challenge. Intriguingly, we found these alterations following LPS challenge were remarkably attenuated by glycine administration, indicating the protective effect of glycine on lung injury. 

NF-κB is a crucial transcriptional factor whose function is associated with the biosynthesis of pro-inflammatory cytokine [33]. To investigate the potential involvement of NF-κB signaling in the beneficial effects as observed, Western blotting was conducted and the result showed that glycine pre-treatment abrogated LPS-induced activation of NF-κB, indicating a regulatory effect of glycine. It is well-known that TLR4/MYD88 is responsible for the upregulation of NF-κB signaling in response to bacterial infection in various conditions [33]. However, this was not the case in our study, because the protein abundance of TLR4 and MYD88 was not affected by LPS. In vivo and in vitro studies have shown that the phosphorylation of p38MAPK is another protein responsible for NF-κB-dependent inflammatory responses and cytokines secretion [34,35,36]. Further study is needed to answer whether p38MAPK is implicated in and contributes to this protective effect.

Recent studies have shown that the activation of the NLRP3 inflammasome is a critical mediator responsible for the maturation of IL-1β for the development of acute lung injury [8,9]. This effect was validated in our study as shown by the enhanced protein levels of NLRP3, ASC, cleaved caspase1, and increased IL-1β. Importantly, these effects were attenuated by glycine administration, indicating a regulatory effect of glycine in LPS-challenged mice. The accumulation of ROS has been reported to activate the NLRP3 inflammasome and downstream targets, including caspase1 and IL-1β, one of the main cytokines associated with lung injury in both clinical patients and experimental animals [12]. Glycine has been reported to alleviate ROS-induced cellular damage by promoting the synthesis of glutathione (GSH), an endogenous antioxidant, in intestinal porcine epithelial cells and other tissues [21,37]. Glycine supplementation might reduce the ROS level due to increased GSH product, therefore contributing to reduced NLRP3 inflammasome and decreases the protein level of IL-1β in the lung tissues.

NRF2 is a transcriptional factor associated with survival by upregulating downstream targets, such as HO1, NQO1, and GSTA4 in response to various stresses [13]. Deficiency of NRF2 signaling leads to severe lung injury of mice in response to LPS exposure [38]. In contrast, enhanced protein levels of NRF2 have been reported to alleviate LPS-induced lung injury in mice [39,40]. In consistent with previous report [41], we found that glycine pre-administration reversed LPS-induced depletion of NRF2 and led to the increased protein level of genes implicated in cellular survival [42]. In a recent study, the authors showed that NRF2 is a repressor of NF-κB [43]. Our results indicated that the glycine-induced downregulation of NF-κB was mediated, at least partially, via NRF2 signaling. Heat shock proteins, including HSP40 and HSP70, are negative regulators of ROS generation [44]. HSP70 knockout mice displayed the excessive activation of NF-κB and increased inflammatory cytokine in the lung tissues [45]. In our study, glycine administration prevented the LPS-induced downregulation of HSP70 and HSP40 to both protein and mRNA level, which might contribute to reduced lung injury through repressing inflammatory responses and inhibiting apoptosis of epithelial cells in the lung tissues, as previously reported [46].

Glycine is traditionally regarded as a nutritionally non-essential amino acid due to its de novo synthesis from serine, choline, threonine, and Glyoxylate [47,48]. Critical roles of glycine on tissue repair, metabolic regulation, and anti-oxidative capacity are recognized in recent years [23,48]. In the present study, we observed a protective effect of glycine on acute lung injury in mice by regulating NLRP3 inflammasome signaling pathway, and survival proteins, including NRF2, HSP40, and HSP70. This is the first study that linked glycine and NLRP3 in lung disorder. Glycine receptor (GlyR), a glycine-gated chloride channel, has been identified in postsynaptic membranes, hepatic and alveolar macrophages, neutrophils, and lymphocytes [49]. Importantly, glycine prevents LPS-induced endotoxemia by activating the GlyR in Kupffer cells [49] or hepatic parenchymal cells [50]. However, both LPS and glycine had no effect on the protein abundance of GlyR, thus excluding an involvement of GlyR and its contribution to the anti-inflammatory effect as observed in the present study. 

The dysfunction of endoplasmic reticulum and activation of autophagy have been reported to implicate in lung injury in both humans and animals [51]. However, we found that LPS treatment did not affect the protein levels of autophagy markers, including P62, ATG5, LC3, and Beclin1, as well as protein levels of activating transcription factor 6 (ATF6), inositol-requiring enzyme 1α (IRE1α), and PKR-like ER kinase (PERK)—well-known sensors for endoplasmic reticulum stress signaling. Therefore, both ER stress and autophagy are not related to the protective effect of glycine. More studies are required to uncover the underlying mechanisms in the future, which might advance our understanding of the benefits of glycine in the context of lung injury.

## 5. Conclusions

In conclusion, we found that glycine administration ameliorated aerosolized LPS-induced acute lung injury, as shown by increased mucin, decreased protein level of pro-inflammatory cytokines. This beneficial effect of glycine was associated with the modulation of the NLRP3 inflammasome and NRF2 signaling. Our findings provide a novel nutritional strategy for acute lung injury by providing glycine to animals. It should be noted that well-defined studies on efficacy and safety are required prior to its application in humans.

## Figures and Tables

**Table 1:** Primer sequences used in Real-time PCR.

Gene	Forward Primer (5’ to 3’)	Reverse Primer (5’ to 3’)
*Muc5ac*	GCAATCCCCTTTCCGATGTC	AAAAGGGCAGGTCTTCGGTA
*Muc5b*	GGTTGGCTACATCTTCTGCG	ATCAGCCCAAATCGCACATC
*Nrf2*	TCCATTTACGGAGACCCACC	GGCCGTTCTGTTTGACACTT
*Ho1*	CAGGTGTCCAGAGAAGGCTT	GCTTGTTGCGCTCTATCTCC
*Nqo1*	GTAGCGGCTCCATGTACTCT	AGGATGCCACTCTGAATCGG
*Gsta4*	TTTAATGGCAGGGGACGGAT	TGTCAGCATCATCCCATCGA
*Hsp40*	TACACATTCCACGGAGACCC	TGAAGCCACCCATACCCATT
*Hsp70*	CAACGTGCTCATCTTCGACC	GGCTGATGTCCTTCTTGTGC
*Gapdh*	AAGCCCATCACCATCTTCCA	CACCAGTAGACTCCACGACA

## Abbreviations

ASC, apoptosis-associated speck-like protein; ATG5, autophagy related gene 5; CON, control group; Gly + LPS, glycine + LPS co-administration group; GM-CSF, granulocyte-macrophage colony-stimulating factor; GSTA4, glutathione s-transferase α 4; HO1, heme oxygenase 1; HSP40, heat shock protein 40; HSP70, heat shock protein 70; IFN-β, interferon-β; IFN-γ, interferon-γ; IL-1β, interleukin-1β; LPS, lipopolysaccharide; LC3, microtubule-associated protein light chain 3; *Muc5ac*, *mucin 5ac*; *Muc5b*, *mucin 5b*; MYD88, myeloid differentiation factor 88; NF-κB, nuclear factor κB; NLRP3, nucleotide binding domain (NOD)-like receptor protein 3; NQO1, NAD(P)H dehydrogenase quinone 1; NRF2, nuclear factor (erythroid-derived 2)-like 2; p-P65, phosphor-P65; PBS, phosphate buffer saline; qRT-PCR, quantitative real-time polymerase chain reaction; RIPA, radio-immunoprecipitation assay; ROS, reactive oxygen species; TLR4, toll like receptor 4; TNF-α, tumor necrosis factor-α
